# Using real-time ascertainment rate estimate from infection and hospitalization dataset for modeling the spread of infectious disease: COVID-19 case study in the Czech Republic

**DOI:** 10.1371/journal.pone.0287959

**Published:** 2023-07-13

**Authors:** Lenka Přibylová, Veronika Eclerová, Ondřej Májek, Jiří Jarkovský, Tomáš Pavlík, Ladislav Dušek

**Affiliations:** 1 Department of Mathematics and Statistics, Faculty of Science, Masaryk University, Brno, Czech Republic; 2 RECETOX, Faculty of Science, Masaryk University, Brno, Czech Republic; 3 Institute of Biostatistics and Analyses, Faculty of Medicine, Masaryk University, Brno, Czech Republic; 4 Institute of Health Information and Statistics of the Czech Republic; University of California San Francisco, UNITED STATES

## Abstract

We present a novel approach to estimate the time-varying ascertainment rate in almost real-time, based on the surveillance of positively tested infectious and hospital admission data. We also address the age dependence of the estimate. The ascertainment rate estimation is based on the Bayes theorem. It can be easily calculated and used (i) as part of a mechanistic model of the disease spread or (ii) to estimate the unreported infections or changes in their proportion in almost real-time as one of the early-warning signals in case of undetected outbreak emergence. The paper also contains a case study of the COVID-19 epidemic in the Czech Republic. The case study demonstrates the usage of the ascertainment rate estimate in retrospective analysis, epidemic monitoring, explanations of differences between waves, usage in the national Anti-epidemic system, and monitoring of the effectiveness of non-pharmaceutical interventions on Czech nationwide surveillance datasets. The Czech data reveal that the probability of hospitalization due to SARS-CoV-2 infection for the senior population was 12 times higher than for the non-senior population in the monitored period from the beginning of March 2020 to the end of May 2021. In a mechanistic model of COVID-19 spread in the Czech Republic, the ascertainment rate enables us to explain the links between all basic compartments, including new cases, hospitalizations, and deaths.

## Introduction

In mathematical epidemiology, compartmental models of type SIR or SEIR are widely used to describe and explain outbreaks of epidemics [1–8]. The classical SEIR models monitor compartments (i) S Susceptible individuals (those who have not yet been infected by the disease and may become so), (ii) E Exposed individuals (those in the incubation period), (iii) I Infectious individuals (those able to spread the disease) and (iv) R Recovered/Removed individuals (those who cannot become infectious anymore; they are either recovered or deceased). Application of SEIR-type models to the COVID-19 pandemic is problematic due to the existence of a non-observable variable of infectious that spread the virus asymptomatically. This problem was studied even before SARS-CoV-2 emergence, and an additional cohort was introduced to model respiratory infection outbreaks, such as the first SARS outbreak in 2002–2003 [9, 10]. These SEIAR models are modifications of the standard SEIR models. Papers [9, 10] provide valuable information on their derivation, *R*_0_ computations, and other related topics. In Supplement, you will find a basic comparison of the SEIR and SEIAR models and the derivation of *R*_0_ for the SEIAR model. The main issue addressed in the manuscript is the estimation of the unreported A compartment.

In the real-world data, positive subjects are not necessarily symptomatic and, on the contrary, not all symptomatic subjects are detected. Without knowing what part of the epidemic is observed, we cannot rely on case reports. This detected part of the infectious individuals is known as the ascertainment rate (AR) and it has to be estimated. Although the compartmental approach is widely used for modeling COVID-19 epidemics in a broad modeling community, most of the time the observational layer estimation is missing [11, 12], or if an undetected infectious compartment is part of such a model, the AR is calibrated as an unknown parameter along with other parameters, as demonstrated in several other studies [13–15]. Information about both the observed and unobserved proportions of the epidemic is crucial for estimating predicted admissions to hospitals and setting up effective and timely interventions [16]. Of course, rough estimations can be performed and used as a fixed value for a long period [17], but changes in the testing and tracking strategy may significantly affect the volume of the observed epidemic and lead to biased estimates of disease spread.

Excess deaths certainly provide the most accurate information about AR, but their usage is possible only retrospectively. Similarly, the case fatality rate (CFR) is unsuitable for modeling an ongoing epidemic in real-time due to the long delay from infection to death [13, 18]. Another option to estimate AR is to use virological and serological participatory surveillance, but again it cannot be done in real time and must also be done retrospectively. According to some estimates [19], only 31% of people with symptoms similar to COVID-19 sought medical attention in a monitored period; such results were confirmed by serological studies [20].

Here, we offer an approach to AR estimation that can be used both retrospectively and in real time. We demonstrate the applicability of our approach of real-time AR estimate on data from hospital admissions due to SARS-CoV-2 infection in the years 2020–21 and show our results on a specific model. In SEIR models, the number of susceptible individuals determines the dynamics of the epidemic, so the peak is primarily driven by the rate of connectivity and mixing. SEIR-type models can still be used if there are reasonably good estimates of the factors influencing the transmissibility rate (related to mixing, environmental variables, etc.), but obtaining this information is difficult when a new virus emerges or when new control interventions are introduced. Therefore, we used modified ZSEIAR model in our case study of COVID-19 in the Czech Republic that uses the dependence of transmissibility purely on mobility data, and all other dependencies such as environmental or social variables are moved to be optimized by feeding Z to S, which replaces social network connectivity or temperature dependence or other unknown variables to estimate the driving force of real-world epidemics. The model is described in the Supplement in detail and is enclosed as an R script.

Another challenging issue is that even studies of the early pandemic period monitoring the almost immune-naive population showed that mild cases of COVID-19 are significantly understated because the severity of symptoms of COVID-19 is age-dependent [21, 22]. We will therefore address the age-dependency of our AR estimate in the paper as well.

## Research methods

### Ascertainment rate independent estimation principle

The basic principle for the estimation of the moving AR estimate is based on the Bayes rule for conditional probabilities and on the assumption that the average probability of hospitalization of an infected person *P*(*H*) at time *t* is given or estimated. We also derive an estimation procedure depending on the age structure.

We will use the following notation:

(P1) *P*(*Det*|*H*)—the probability that a person admitted to a hospital for the infectious disease was previously detected at time *t*; to estimate this probability, we use the 7-day or 14-day moving proportion of patients reported before admission to hospital from all patients hospitalized for the infectious disease (including those not detected before admission to hospital) with respect to the date of the positive test report
(P2) *P*(*H*|*Det*)—the probability that if an individual was detected at time *t*, he or she would be hospitalized; to estimate this probability, we use the 7-day or 14-day moving proportion of all reported hospitalized patients detected before admission to the hospital, from all that time already detected subjects with respect to the date of the positive test report, that is, except those detected in the hospital afterward who are part of the undetected compartment at time *t*
(P3) *P*(*H*)—the probability that an infected individual is / was / will be hospitalized for the infectious disease (irrespective of whether it was detected or not) at time *t*; should be derived for each community/country separately since it is highly dependent on the structure of the age of the population, a possible estimation method is described below

We can calculate the estimation of *p*(*t*) = *P*(*Det*) as the moving average relative to the date *t* of the positive test report according to the Bayes formula

$$\begin{array}{c} p\left(t\right)=P\left(Det\right)=\frac{P(Det|H)P(H)}{P(H|Det)}. \end{array}$$ (1)

Therefore, to summarize the principle of estimation, we estimate the invisible part of the epidemic using knowledge about late-detected individuals (i.e., undetected infectious subjects) who end up in hospitals with a severe symptoms of the infectious disease and who are confirmed there afterward. At least a 7-day window has to be used for the moving average due to the week oscillations. A 14-day window is possible in case there is a low number of hospitalizations since a low number of hospitalizations results in greater variability of all estimates. On the other hand, 14 days moving average flattens the curve also in case of sudden changes, which can hide early information about the outbreak.

An unknown value in the Bayes formula (1) is the specific probability of hospitalization *P*(*H*), which needs to be estimated, for example, from surveillance studies [23–25]. Other methods can also be used without an additional surveillance data set: (1) infection-fatality rate (IFR) and hospitalization-fatality rate (HFR) estimates, since the average probability of hospitalization due to the infection is HFR/IFR, (2) nationwide non-indicated antigen test screening data with comparison to hospitalized and observed infectious cases or retrospectively also (3) excess deaths data. All these methods are presented in the Results section for the data on the COVID-19 epidemic in the Czech Republic. These indirect pieces of evidence support our estimate of the average probability of hospitalization due to COVID-19 infection for the Czech population in the monitored period. The approach can be adapted by analogy for use in another community/country.

Because the probability of hospitalization due to an infectious disease is usually age-dependent, we propose a method based on a rough estimate of the relationship between total and age-specific probabilities of hospitalization and the age structure of the monitored population divided into *n* age categories, that is

$$\begin{array}{c} P\left(H\right)=\sum_{i=1}^{n}w_{i}P\left(H_{i}\right), \end{array}$$ (2)

where *w*_*i*_ ≥ 0, satisfying $\sum_{i=1}^{n}w_{i}=1$, are proportions of the age-structured monitored population, and *P*(*H*_*i*_) are the age-specific probabilities of hospitalization due to infection for given *i* = 1, …, *n*. In case of COVID-19, the minimum necessary division of the weighted average is into two groups: non-seniors (65-, i.e. individuals under 65) and seniors (65+, i.e. individuals over 65).

### Necessary data for real-time AR estimation

To estimate AR in real time, it is necessary to continuously collect both hospital data and laboratory data. The minimum personal record must include age or age group, date of positivity, and if hospitalized, then date of admission to the hospital due to the infectious disease.

In the Czech Republic, data on reported SARS-CoV-2-positive individuals and COVID-19 patients and their hospital stays are collected and processed by the Information System of Infectious Diseases (ISID) almost in real-time [26]. ISID includes a complete health care information record for a person; we used a dataset (shared at [27] that includes variables describing the infection case: district and regional number, sex, age group (0–19, 20–64, over 65), date of the first symptom, date of sampling collection, date of a positive result, date of report, date of isolation, date of admission to a hospital, end of hospitalization, date of recovery, date of death.

An unspoken but important assumption is that all hospital patients are tested for the infectious disease. In case of a positive test, the result should be entered into the data collection system even if the patient had not tested positive before admission. However, this is common practice in most developed countries. In the case of COVID-19, persons requiring hospital health care with severe respiratory problems are almost all tested for SARS-CoV-2 immediately after admission.

### Ascertainment rate usage in modeling epidemics

**Fig 1:**
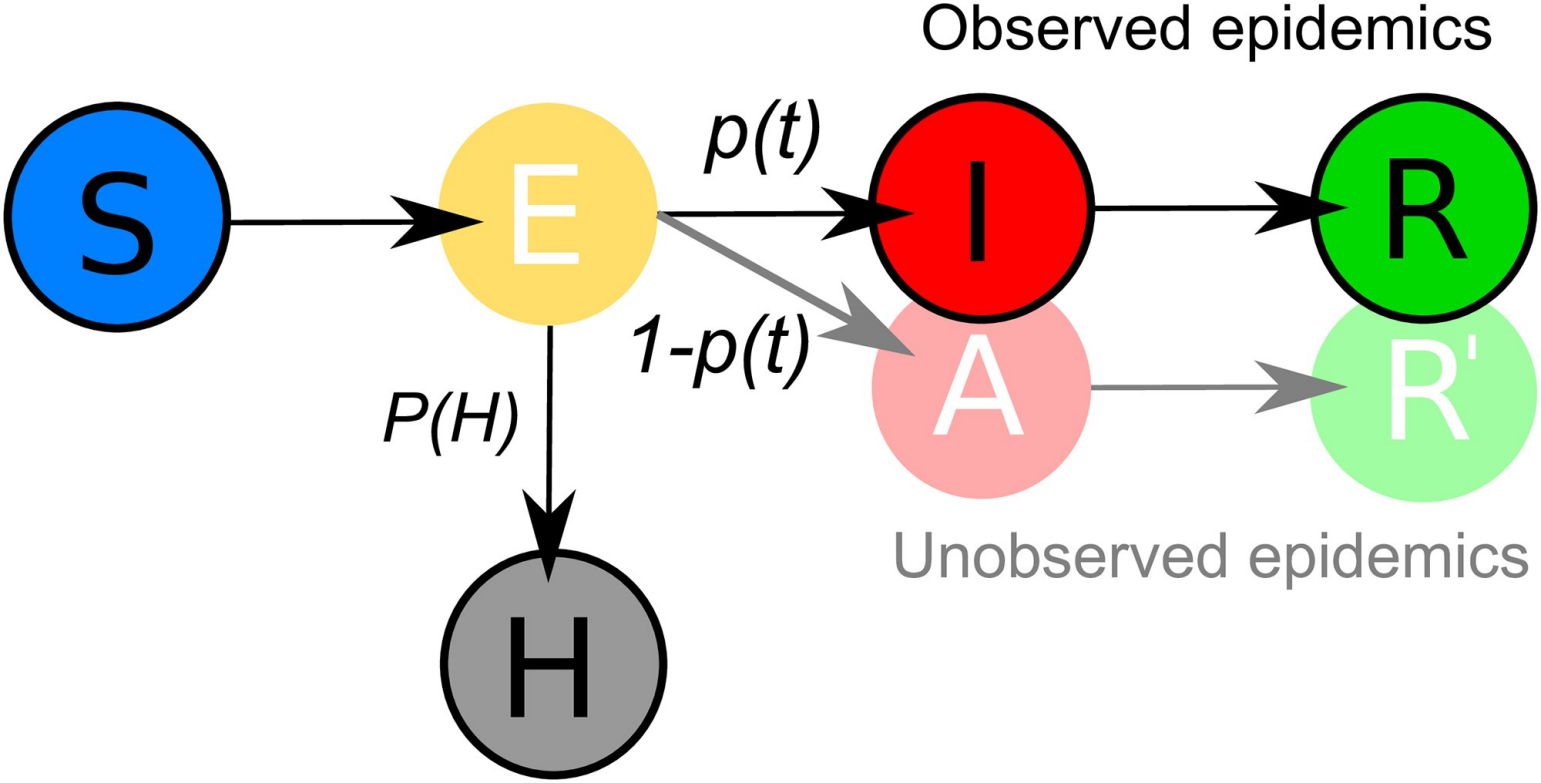
SEIAR model scheme. The compartment *H* of hospitalized patients due to infectious disease is recruited from the exposed compartment *E* with a given probability *P*(*H*). We can estimate the size of the exposed compartment using the estimation of the ascertainment rate *p*(*t*) at time *t* from the reported epidemic (i.e., the observed cases *I*), while the compartment *A* remains unobserved.

SEIR-type models are commonly used for epidemic monitoring, primarily to predict the number of severe cases that require hospital care. It is common practice to assume a fixed proportion of observed infectious or hospitalized individuals during the course of an epidemic, unless new drugs are discovered, the population structure changes significantly, or other major epidemiological factors come into play. The compartment of hospitalized subjects H is usually calibrated through observed epidemics as its fixed part [17, 28–30]. Using accurate real-time data to estimate AR *p*(*t*) can improve epidemic and hospitalization surveillance. Fig 1 shows a scheme of the classical SEIR/SEIAR model with the observed (bright colored circles with solid border) and unobserved (pale colored circles without border). Here, *P*(*H*) is the probability of hospitalization due to an infectious disease, as discussed in the previous subsection.

## Results

Our main result is a novel method to estimate AR and its changes that can be used in real time. To show this method’s successful use, we present a case study of the COVID-19 epidemic based on data from monitored period from March 2020 to May 2021 in the Czech Republic, where we used this real-time estimation in a model that is described in detail in S1 Appendix. The model code in R is also included.

### Probability of hospitalization due to SARS-CoV-2 infection—Derivation of the age-structure dependence

In our case study on the Czech population, the probability of hospitalization due to SARS-CoV-2 infection described by formula (2) can be simplified using the minimum division into non-senior and senior population parts as

$$\begin{array}{c} P_{CR}\left(H\right)=\frac{4}{5}P\left(H_{65-}\right)+\frac{1}{5}P\left(H_{65+}\right), \end{array}$$ (3)

since about 1/5 of the population is over 65 years old in the Czech Republic [31]. On the contrary, the majority of hospitalized patients were in the elderly population. The anonymized dataset [27] from ISID used for real-time COVID-19 monitoring contains information about age. There are three age groups: those aged 0 to 19 years (0–19y) and 20 to 26 years (20–64y) will be referred to as non-senior or 65- population, whereas those aged 65 years or older will be referred to as senior or 65+ population. The long-term proportion of the hospitalized population over 65 was 3/4 resulting in a risk ratio of 12 for the senior versus the non-senior population. Similarly, a rough estimate of the age-dependent hospitalization risk ratio can be made for other data sets.

This rough estimate is also consistent with the log-linear relationship between the infection fatality rate (IFR) and age, as published in a study by Levin et al. [32] and our recent analyses of Czech data [33, 34]. Using this estimate and the known age structure of the reported infectious individuals, we obtain a rough estimate of the time-dependent probability of hospitalization that varies according to the age of the infected

$$\begin{array}{c} P\left(H\right)=p_{65-}^{+}P\left(H_{65-}\right)+p_{65+}^{+}P\left(H_{65+}\right)=(1+11p_{65+}^{+})P\left(H_{65-}\right), \end{array}$$ (4)

where $p_{65+}^{+}$ and $p_{65-}^{+}$ are 7-day moving averages of the senior and non-senior (children included) population ratio in the reported cases. We obtained the dependence of the probability of hospitalization on the ratio of the infected senior population.

Note that it is necessary to use some average level of probability of hospitalization due to COVID-19 infection in the Czech Republic *P*_*CR*_(*H*). This level can be estimated using various methods based on indirect evidence, but high precision is not important for short- and medium-term predictions of hospital admissions. However, a good estimate of this average level of hospitalization probability is necessary for long-term forecasts and herd immunity estimates. In the following, we present the options that can be used to estimate the average level of hospitalization probability. For the Czech Republic, we used $P_{CR}\left(H\right)=\frac{1}{50}$, which is employed later on. Average *P*_*CR*_(*H*), estimations (3) and *P*(*H*_65+_) = 12*P*(*H*_65−_) imply formula (4) in the form

$$P\left(H\right)=\frac{1}{160}\left(1+11p_{65+}^{+}\right).$$

The method based on HFR and IFR ratio must rely on data from countries where contact tracing was performed more thoroughly. Countries such as South Korea, Malaysia, Thailand, and Singapore showed an observed case-fatality ratio 0.5% [35–38], which is assumed to be close to the IFR [18, 22, 32, 39, 40]. Our estimate $P_{CR}\left(H\right)=\frac{1}{50}$ together with an average of 22% HFR (from MZ CR data [27]) during autumn 2020 gives IFR of 0.44%.

The estimate of the average probability of hospitalization due to the infection can be supported by surveillance studies, or we can use wide non-indicated or other representative testing data as full-scale testing of employees, schoolchildren, and students. The nationwide non-indicated antigen test screening in the Czech Republic showed an average test positivity of 5% in December 2020. This is another strong supporting argument for estimating the probability of hospitalization as $P_{CR}\left(H\right)=\frac{1}{50}$ since the consequence of this *P*_*CR*_(*H*) level was the estimation of AR around 0.25, and half a million infected active cases fully corresponds to the observed quarter of 120,000 active cases at that time.

**Fig 2:**
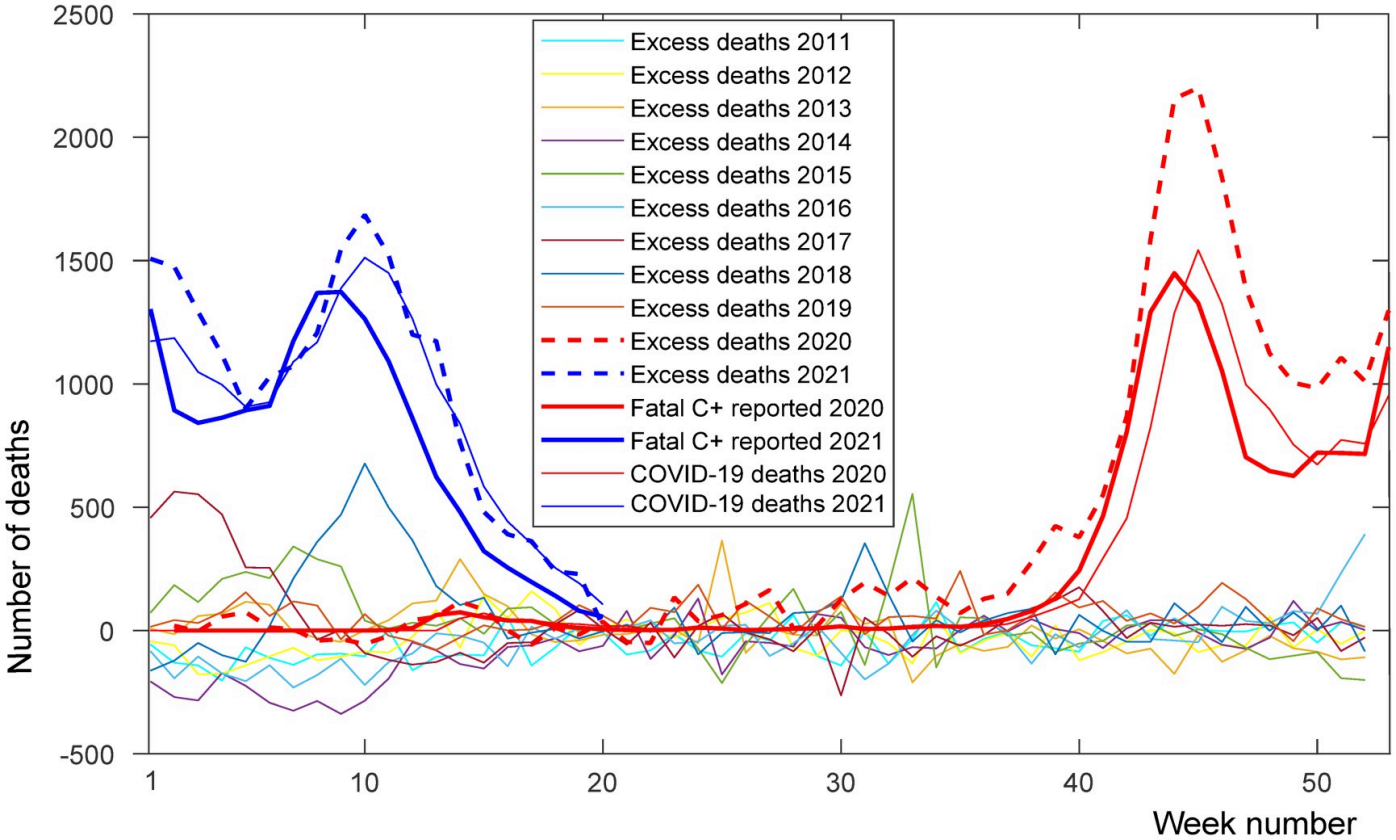
Excess deaths in the Czech Republic. Excess deaths in weeks of the years 2020 (red thick dashed line) and 2021 (blue thick dashed line) according to average week deaths in years 2011–2019 (thin solid lines show excess deaths in weeks of the years 2011 to 2019) [41], weekly reported SARS-CoV-2 positive subjects that died (red and blue thick solid lines), and weekly reported deaths (red and blue thin solid lines) [27]. Our model works with data related to the date of the report, including fatal reports (thick line), and excess deaths (dashed line) and COVID-19 deaths (thin line) are related to the day of death.

Retrospectively, excess mortality can be used. In the Czech Republic, excess mortality during autumn and winter 2020 (from [41]) is highly correlated with the epidemic wave and gave approximately 0.5 excess unreported deaths to each reported patient who died with COVID-19 (using an anonymized dataset created from ISID data that can be downloaded from the MZ CR portal [27]), see Fig 2.

Since about half of the hospitalized were detected only after admission to a hospital (which implies that half of them were not reported during the infectious period), meaning that seriously ill people were detected late, we can deduce that the observed part of the infectious compartment could be around 33% at the beginning of the year 2021 which corresponds to the AR estimate at that time and is also in agreement with a study published by Pullano et al. [19].

**Fig 3:**
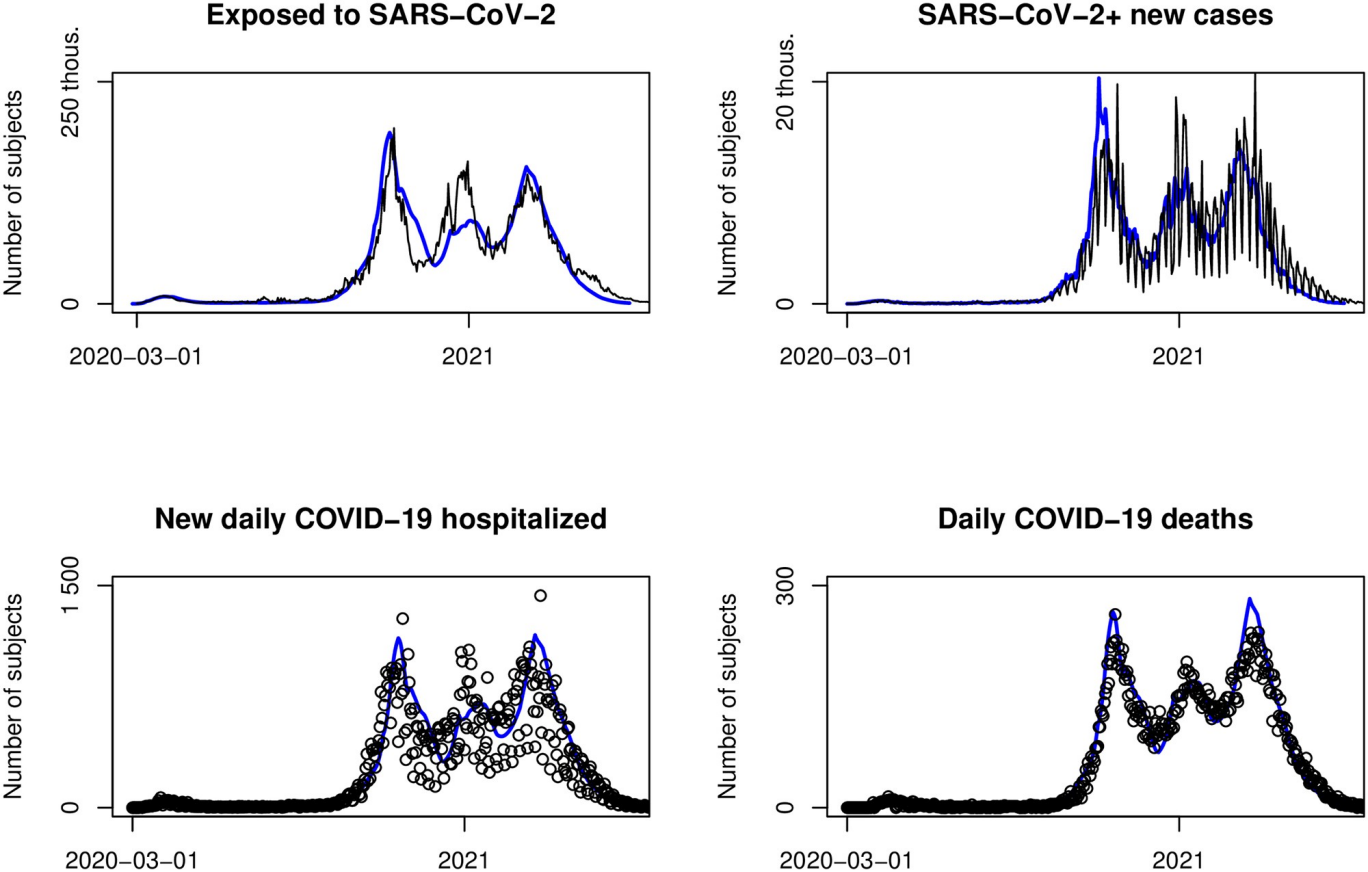
Epidemic monitoring. Exposed (new cases divided by AR estimate), new cases, hospital admissions and daily deaths (black), and model fit optimized to hospital admissions (blue).

An improvement can be made in the age structure dependence of the probability of hospitalization. We improved it to depend on three age groups from May 2021. Until then, the probability of hospitalization in the Czech Republic was distinguished only between two age groups (under/over 65), but this started to be non-sufficient since COVID-19 spread across younger people and mandatory testing has been introduced in schools. The children were hardly tested before due to the age specificity of the symptoms. Retrospective data analysis shows that people under 20 were hospitalized with almost zero probability and three age compartments (under 20, 20–65, and over 65) appear to be enough for the basic improvement of the *P*(*H*) estimate in the form

$$P\left(H\right)=\frac{1}{160}\left(1-p_{20-}^{+}+11p_{65+}^{+}\right),$$

where $p_{20-}^{+}$ is a 7-day moving average of the under 20 population ratio in the reported cases. The need for this modification is also visible in Fig 3.

### Ascertainment rate estimate usage—Epidemic monitoring

We have been using a compartmental model including AR for epidemic monitoring during the years 2020–21 in the Czech Republic (see S1 Appendix). The initial outbreak is set to the time when the first person in the Czech Republic tested positive for SARS-CoV-2 at the beginning of March 2020. The period described here includes the dominance of wild-type and alpha variants of SARS-CoV-2 until May 2021. Later, we used more complex models of SEIAR and SEIARS types, which included data on vaccinations and reinfections. Fig 3 shows the estimated exposed using real new cases divided by AR estimate, real new cases, real daily admissions to hospitals and deaths (black circles) and the optimized fit to hospitalization data (solid blue line) with the AR estimate average baseline $P_{CR}\left(H\right)=\frac{1}{50}$. You can see here that the model explains all the compartments in the whole period except for a short period of Christmas. This period was burdened with several unknowns. The main one was the arrival of the new alpha variant; in the Czech Republic at that time, there was no surveillance either by variant multiplex PCR method or sequencing, so we could not change the model parameters according to the variants in proper time and proportion. The second unknown was people’s behavior during Christmas time. They postponed hospitalizations more than usual. And the third was a significant change in testing strategy before Christmas—the government introduced free antigen tests for everyone. This should be the minor problem since the estimate is robust against testing strategy changes outside hospitals. Despite these unknowns, the model fits very well.

**Fig 4:**
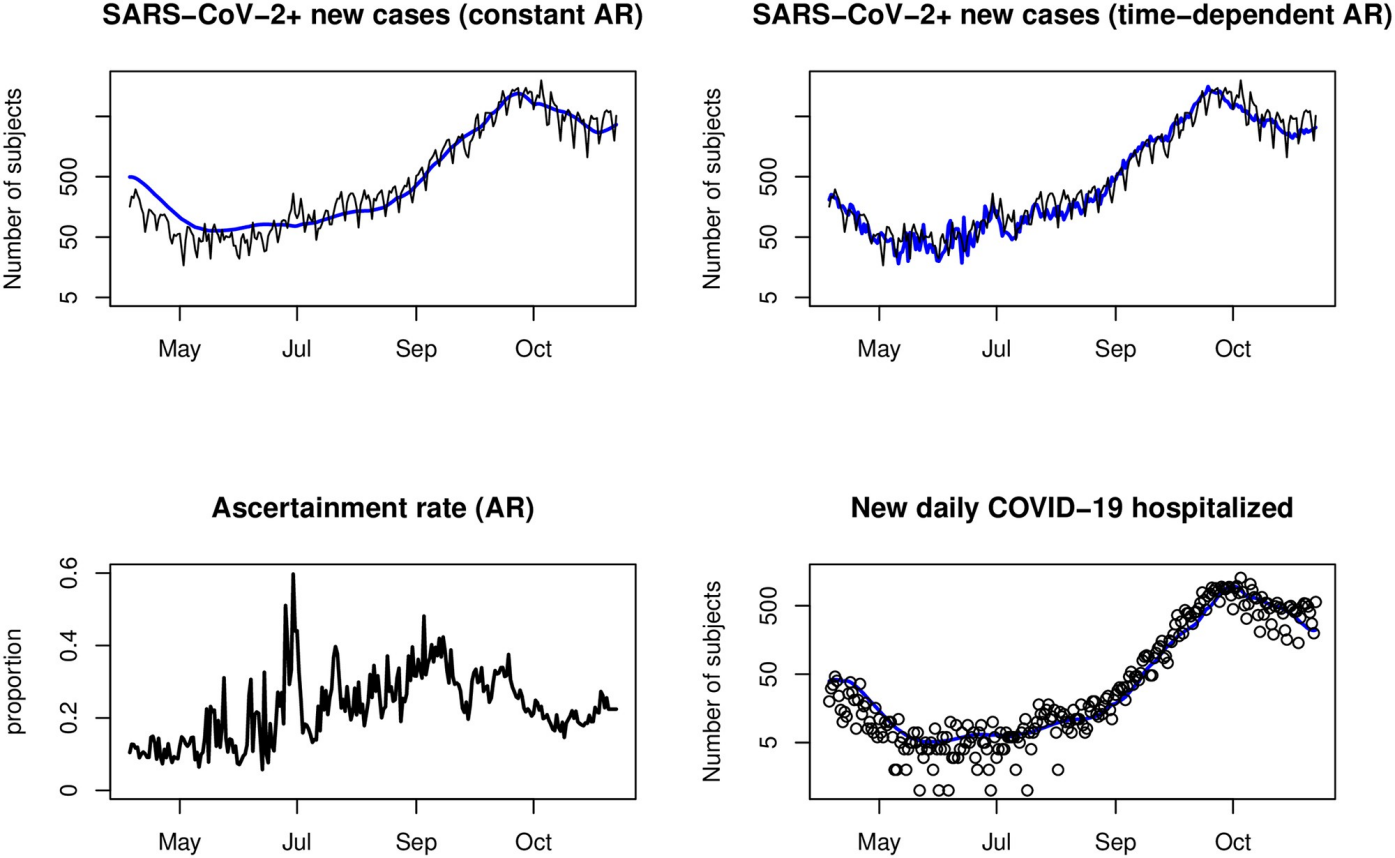
Comparison of using constant AR and moving AR. The model (blue) fitted to the data (black) of new hospitalizations (bottom right) reproduces the actual new cases (top black) only when using moving AR, whereas using constant AR does not account for either the number of cases in the first wave or the fluctuation due to the change in testing during the OKD outbreak (Jul).

We used the AR estimate and the transmissibility rate as strictly dependent on the number of contacts or mobility. Other dependencies were included in the estimate of affected susceptible compartment *S*, which was supplied from an additional fitted compartment. This simulated the first outbreak very well from April 4, 2020, until the summer; see Fig 4. During the first 35 days, the laboratories’ capacity and the testing and tracing system capacity increased substantially. For this period, we could not even estimate the AR due to the small number of hospitalized. We let it constantly grow due to the lack of data at the beginning of the outbreak, since it corresponds to an increase in test capacity in this period, and started to compute the moving average of AR from time *t* = 35 (*t* = 0 marks the first detection day—March 1, 2020). Assuming that the probability that the infected person will be hospitalized is $P_{CR}\left(H\right)=\frac{1}{50}$, we calculated *p*(35)≐0.13 from the data of hospitalized subjects by Bayes formula (1) at the beginning of April 2020.

The usefulness of using moving AR is shown in Fig 3. On a logarithmic scale, it shows the first two waves (the waves differed in scale), including the period of July 2020. During this period, a local outbreak in OKD mines led to mass PCR testing in the whole of OKD mines employees (OKD mines carry out hard coal mining in Karviná part of the Ostrava-Karviná district in Moravian-Silesian region). Since the prevalence of COVID-19 was low in other regions of the Czech Republic at that time, this outbreak enormously changed AR. It is obvious that the model fitted to hospitalizations corresponds to real data of new cases compartments only in the case of using moving AR, while it does not in the case of using the average constant AR (different AR in the two waves visibly led to different CFR or hospitalization rate in the two waves); therefore, the fluctuation due to the change in testing during the OKD outbreak is not reproducible at all.

### Ascertainment rate estimate usage—Early warning

The AR estimate can be used as an early-warning indicator. As we saw in the previous subsection, retrospective analysis provides a good explanation of the OKD wave: new cases did not significantly affect the dynamics of hospitalized patients and deaths (see Fig 4). Similarly, if the increase in the number of new cases is offset by a decline in AR, the outbreak may remain hidden.

**Fig 5:**
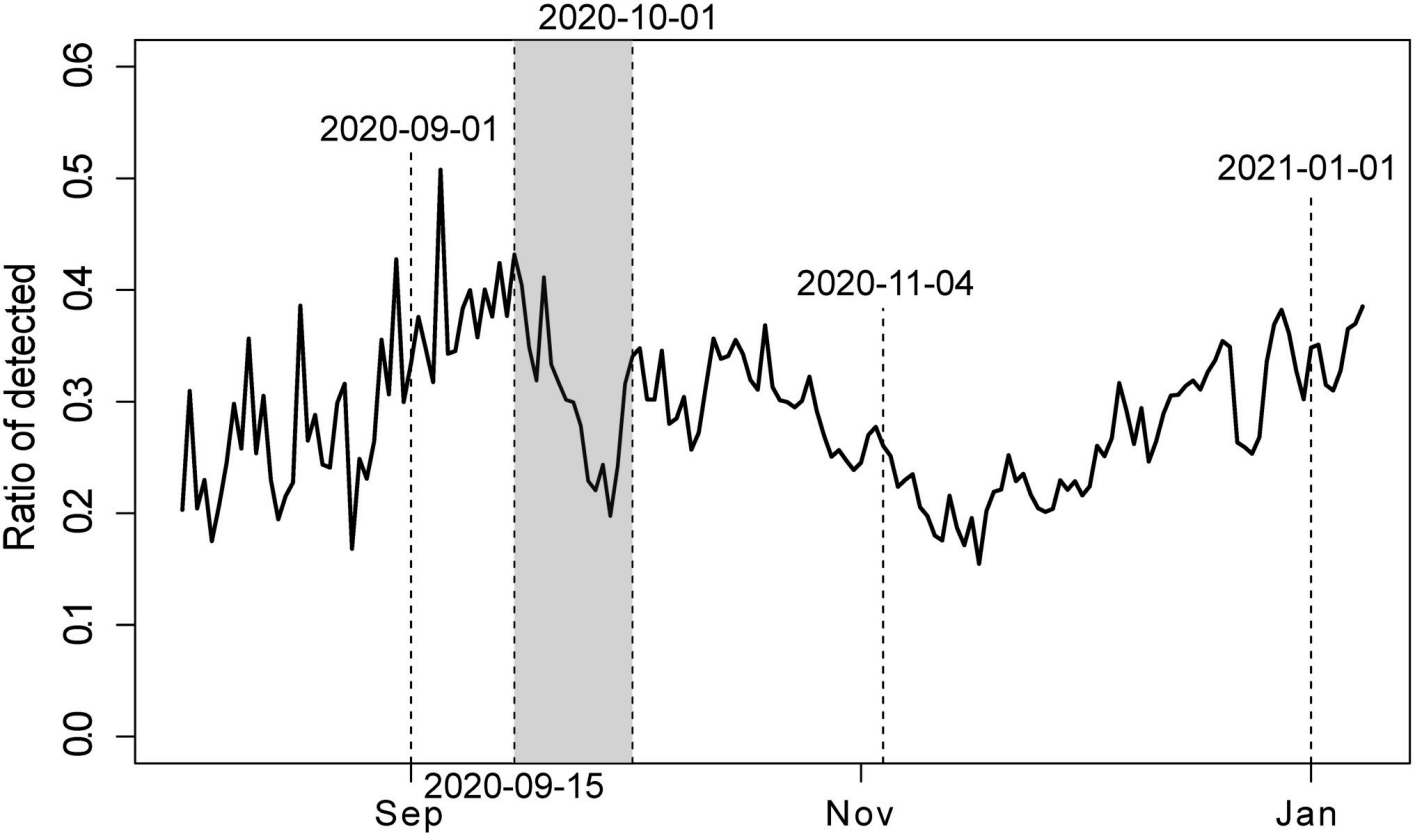
Insufficiency of tracing in the second half of September 2020. The peak of the second wave is marked on 2020-11-04. The dates in the chart denote: 2020-09-01—the beginning of the school year; 2020-09-15 to 2021-10-01—tracing overload; 2021-11-04—peak of the second wave; and 2021-01-01—New Year.

Actually, the AR estimate shows the likely decrease in the effectiveness of tracing by the Regional Public Health Authorities (RPHAs), which occurred in the second half of September (Fig 5, grey period). A significant decrease in the AR estimate was a signal of contact tracing and testing system overload. There are other declines—during the epidemic waves (see Oct-Nov). But these are related to the peak period of the epidemic. The detection rate was limited due to a more or less linear increase in capacities of RPHAs and call centers, but an approximately exponential increase of cases. The September decline before the wave was an early-warning signal.

**Fig 6:**
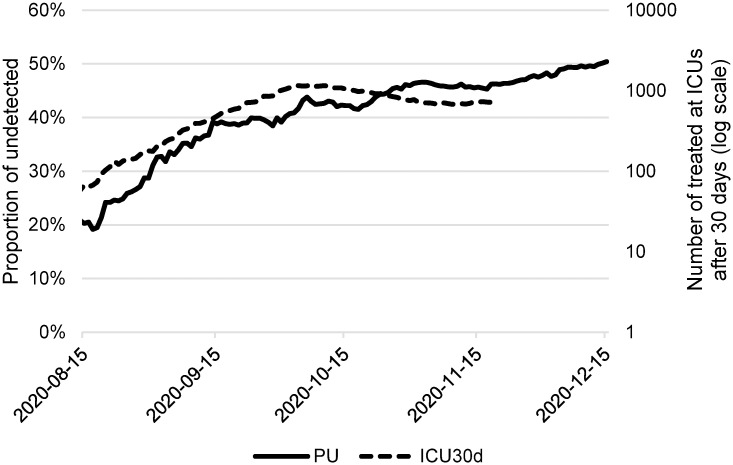
Correlation with ICUs. Proportion of undetected in the community (PU) and number of COVID-19 patients treated in intensive care units after 30 days (ICU30d).

Between mid-September and mid-December 2020, we observed a high correlation of the proportion of newly hospitalized non-detected in the community 1 − *P*(*Det*|*H*) and the subsequent number of COVID-19 patients treated at intensive care units (Fig 6, correlation coefficient 0.92). This confirmed the suitability of this parameter as a good indicator of the future burden of hospital care. Based on the fact that the positivity of the tests changed after the introduction of antigen screening tests (and therefore ceased to be a proxy variable for AR), we instead introduced this indicator to the epidemic Risk Index in the Czech Republic.

### Ascertainment rate estimate usage—Effectiveness of non-pharmaceutical interventions (NPIs)

**Fig 7:**
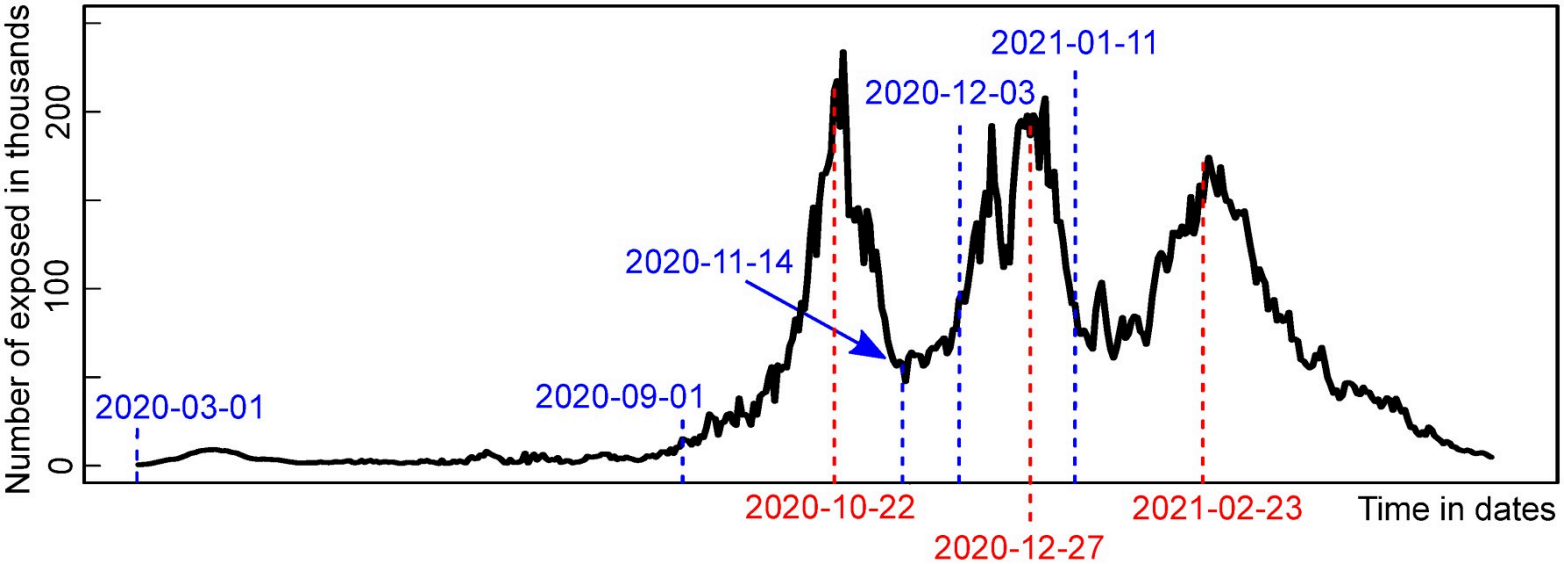
Exposed population and NPIs. Exposed estimated using new cases divided by AR estimate compared to the introduction of NPIs: 2020-09-01 (beginning of the school year), 2020-10-22 (a partial lockdown), 2020-11-14 (announcement of the first measure releases after 2020-11-18), 2020-12-03 (shops reopening), 2020-12-27 (a partial lockdown), 2021-01-11 (partial school reopening), 2021-02-23 (mandatory respirators).

Another possible application of AR is the opportunity to monitor the effectiveness of NPIs retrospectively. By estimating the actual number of infections, it is possible to visualize the data, including the ‘invisible’ part (with a proper delay given by the incubation period and the mean time to the report), and discuss directly how the established NPIs have possibly worked in the Czech Republic. Fig 7 shows several specific dates: 2020-09-01 (beginning of the school year), 2020-10-22 (partial lockdown), 2020-11-14 (announcement of the first measure release, partial school reopening 2020-11-18), 2020-12-03 (shops reopening), 2020-12-27 (a partial lockdown), 2021-01-11 (partial school reopening), and 2021-02-23 (mandatory and widespread introduction of respirators followed with partial lockdown).

Time series for fits to cases, estimated exposed, hospitalized, and deaths are depicted in Fig 2.

## Discussion and conclusion

In the paper, we presented a method for moving AR estimates that can be continuously monitored during the epidemic in real-time. It can also be used retrospectively. The method is based on the Bayes formula and compiling information from independent datasets of positively tested infectious individuals and hospital admissions with a principal diagnosis of the monitored infectious disease.

The main advantage of this method is that the estimate is rigorous and independent, so there is no need for calibration in a compartmental model with undetected compartments. From 2021 onwards, many modeling teams used mechanistic compartmental models as a starting point and developed their own models to monitor the epidemic [42]. Those models are collected on platforms such as the European Covid-19 Forecast Hub [43, 44], coordinated by the European Centre for Disease Prevention and Control (ECDC). European Covid-19 Forecast Hub is coordinated by ECDC, and the models for the ongoing COVID-19 pandemic are collected and monitored at this platform; model performances are evaluated, and ensemble models are made [43, 45]. In some models from the European Covid-19 Forecast Hub, the authors empathize with the role of the observable part of the epidemic (such as the FIAS_FZJ-Epi1Ger team [46], or the DSMPG-bayes team [47]) and use their own methodology to model it. The compartmental approach is used in a broader community than just at ECDC Forecast Hub, but usually either AR estimation is missing [11, 12] or it is calibrated as an unknown parameter [13–15]. To the best of our knowledge, there is no other modeling group that estimates the time-varying observable part of the epidemic independently from hospital data in real time. We are unaware of any other published method of estimating time-varying real-time AR (except using proxy variables that include the proportion of positive tests, unfortunately, sensitive to changes in testing strategies). We submitted predictions with outstanding evaluations [48] since March 2021 at the ECDC Forecast Hub [43], our model continuously showed high-quality predictions for the Czech Republic. The relative measure of our model’s two-week forecast performance called relative weighted interval score (relative WIS [44]) evaluated for the first 30 weeks was the best of all modeling teams (0.37) and even better than the Eurocovidhub-ensemble score (0.42). We believe that if a similar real-time hospital data collection system exists in other countries, the method may significantly refine and simplify many existing models despite the type of infectious disease.

The AR estimate can also monitor the overall increase of infected in the population and real prevalence, so it can monitor possible approaching herd immunity (in case of a low rate of reinfections) and better predict possible scenarios for later epidemic dynamics. For the Czech Republic, this provides indirect evidence that it was very far from approaching herd immunity in summer 2020. When we calibrated and optimized our model with the average level $P_{CR}\left(H\right)=\frac{1}{50}$, we obtained a total number of at most 80,000 overall infected individuals (0.8% of the population) in May 2020 (after the first wave). This estimate is entirely consistent with the SARS-CoV-2-CZ-Preval prevalence study [49] that estimated the range of prevalence values for SARS-CoV-2 antigen positives between 0% and 0.22% in regions where active cases at that time corresponded to 40 positively tested per 100,000 inhabitants and between 0% and 0.4% in regions where active cases at that time corresponded to 140 positively tested per 100,000 inhabitants.

If it is possible to use the method to calculate the AR estimate at the regional level, it can be used to compare the situation in selected regions. A problem for regional use may be the high variability in the case of low numbers of hospital admissions, which can be reduced by extending the moving average window from 7 to 14 days. Supplement elaborates on these limitations and possess an illustrative example with a restriction and comparison of the AR estimate calculation from the whole Czech Republic and the Moravian-Silesian region, enclosed in R code AR_comparison.R. Regional reduction of the data at periods of low disease prevalence implies high variability and inaccuracy of the estimate, whereas at times of outbreak, stratification and calculation for the affected region gives a better estimate for the area. Moreover, assuming that there is no significant difference in the probability of hospitalization in individual regions, we can measure the relative AR by the ratio of regional AR with respect to the reference region. This measure was used as one of the regional tracing effectiveness indicators in the Czech Republic during the epidemic in 2020–2021 in regional weekly reports for key stakeholders.

There are also limitations of the described approach. Some principal limits are related to data collection. First of all, hospitalized subjects’ data must be collected continuously in almost real-time. Data collection may be inaccurate if, for example, hospitals are overwhelmed. In the Czech Republic, there has never been a period in which emergency care was not provided during the COVID-19 epidemic, but during some periods when case numbers peaked, there were delays of several days in reporting. The second limit is the lack of data due to low hospitalizations (for example, during European summer). Extending the moving window for calculations flattens the AR estimate. There is also more significant variability at times of lower hospitalization numbers. However, the technique seems robust to different testing regimens because it relies on unmissable severe cases (and if oxygen support data collection in real-time is available, it can also be used). Various testing regimes were held in the Czech Republic (also screening with antigen tests) during the monitored period, and the method was effective over time.

There are limitations related to the used method based on the estimated fixed average probability of hospitalization due to the disease. Changes in the virulence or other characteristics of viral variants must be considered. The estimate $P_{CR}\left(H\right)=\frac{1}{50}$ is valid for wild-type coronavirus lineages. In 2021, a new variant alpha B.1.1.7 spread in the Czech Republic and dominated. According to studies [50, 51], we increased the transmissibility rate to a 1.5 times higher level during this time in the model; higher average probabilities of hospitalization and death were also to be taken into account. The data show interesting information that the mutation did not affect the ratio of death rates in hospitals between categories 65- and 65+, more precisely *P*(Death|H and 65-)/*P*(Death|H and 65+) ≐ 1/4 during the entire epidemic before the vaccination introduction. Let us note that the ratio then changed to the disadvantage of non-seniors, which can be considered evidence of the effectiveness of vaccines against hospitalization (seniors were prioritized in the vaccination schedule). The unaltered ratio of non-senior and senior death rates implies that the increase in the death rates due to the new variant in both age categories must also be proportional. Our model fits a rough estimate of a 10% increase in the hospital death rate and an approximately 25% increase in the probability of hospitalization during the spring outbreak of a new variant B.1.1.7 in 2021.

Another issue that has to be incorporated is the ongoing vaccination process, which strongly influences the probability of hospitalization. It is a more complicated problem. This paper shows the modeling method on data with the model fixed and conserved in time before summer 2021 (before delta variant B.1.617.2 dominance). In the model whose predictions we continuously published on the ECDC hub until June 2022, we solved this problem in a simplified way, namely by reducing the transmissibility rate and the probability of hospitalization and death proportionally to the percentage of vaccinated people based on effectiveness computed in [33, 34]. Various later recalibrations and minor model changes were continuously specified in the description of the MUNI_DMS-SEIAR model at the ECDC Forecast Hub [43] as they arose over time due to new variants’ emergence or vaccination, etc. The model described in the Supplement is limited to the period when the population was mostly unvaccinated—not because the AR estimation method could not be used, but because of the chosen model. The effects of vaccination and its waning change the probability of hospitalization of the vaccinated, and the models we used further were more complicated. We wanted to show the usage of AR in various ways in the most simple but realistic model. The lineages from the delta variant B.1.617.2 dominance period are not included in this paper for the same reason.

Challenging issues arise with lineages evolving from the omicron variant, where the probability of hospitalization dropped significantly. The omicron variant is really a game-changer that shows an increase in the number of cases included in hospitalization dataset that are “with” covid, and not “for” covid when it is the principal diagnosis. Patients hospitalized for COVID-19 generally need some respiratory support, so if there is enough data, those data can be used instead of only hospital admissions data. In our data set, we also have the date of the oxygen support recorded. However, patients usually arrive in a serious condition with a delay, so it is more convenient to work with hospital admission data. An open dataset [52] shows that during the analyzed period 2020–21, the proportion of the most severe cases (ICU need) was very stable. That also justifies using hospital admissions data in the Czech Republic in the monitored period. That is not true for the omicron variant period. Moreover, the level of reinfections became significant during the omicron variant dominance period. In that case, the SEIARS-type model had to be used instead of the SEIAR-type model.

Another issue that arises from using AR in the SEIR-type model using data is related to data stratification. In the case of the Czech Republic, stratification is not necessary and our approach can be applied without stratification due to the relatively small size of the country and the homogeneity of population density. However, in the case of countries with varying population densities, stratification may be necessary. Our approach can be easily adapted to include such stratification, by using region-specific AR estimates, mobility data or other relevant variables to capture the spatial dynamics of the epidemic.

We believe that a moving estimate of AR is essential for monitoring the ongoing epidemic, and our approach brings a credible estimate in almost real time. We hope that our results will be helpful both for the modeling community in other countries and for further research in the field, as many countries collect data from hospitals [53].

## Supporting information

S1 Appendix Supplement. (PDF)

S1 Data Data and R code. (ZIP)
