# Supplementary material for: Using real-time ascertainment rate estimate from infection and hospitalization dataset for modeling the spread of infectious disease: COVID-19 case study in the Czech Republic
Source: PLoS One. 2023 Jul 13;18(7):e0287959. doi: 10.1371/journal.pone.0287959 (PMC10343065; doi:10.1371/journal.pone.0287959)
Supplement: S1 Appendix — (PDF) [file pone.0287959.s001.pdf]

SUPPLEMENTARY MATERIAL

ZSEIAR model of COVID-19 spread in the Czech Republic using real-time ascertainment rate estimate from infection and hospitalization dataset

Lenka Přibylová [1*], Veronika Eclerová [1,2]

1 Department of Mathematics and Statistics, Faculty of Science, Masaryk University, Brno, Czech Republic
2 RECETOX, Faculty of Science, Masaryk University, Kotlarska 2, Brno, Czech Republic

* pribylova@math.muni.cz

Compartmental epidemic model

General model SEIAR with ascertainment rate estimate

The ascertainment rate (AR) estimate makes it possible to extend the basic SEIR model for the COVID-19 epidemic by adding a compartment A of undetected subjects. In our model, A stands for absent rather than asymptomatic. The definition of an asymptomatic carrier is disputable in real time (the carrier may be presymptomatic), hardly observable, but, unfortunately, carriers with no symptoms are likely to be infectious [1–4]. It is very likely that we do not detect a considerable part of the asymptomatic cohort, but at the same time, it is not true that we detect the entire symptomatic cohort [5]. However, the observed part of the epidemic, which we see in positively tested and reported subjects, is necessarily only a part of the real dynamic epidemic process. Therefore, it is more advantageous to divide the infectious compartment (I) in the basic SEIR model into two parts: I detected (reported) infectious and A undetected (absent) infectious regardless of the presence of symptoms. Compartments S, E and R are susceptibles, exposed, and removed, respectively. The dot indicates differentiation with respect to time.

$$
\begin{aligned}
\dot{S} &= -\beta S(I + A),\\
\dot{E} &= \beta S(I + A) - \gamma E,\\
\dot{I} &= \gamma p E - \mu_1 I,\\
\dot{A} &= \gamma(1 - p)E - \mu_2 A,\\
\dot{R} &= \mu_1 I + \mu_2 A,
\end{aligned}
\tag{1}
$$

This SEIAR model is commonly assumed to be normalized, that is, $S + E + I + A + R = 1$, the parameter β is the transmissibility rate of SARS-CoV-2, $1/\gamma$ is its latent incubation period, $1/\mu_i$, $i \in \{1, 2\}$ are the mean infectious periods in each infectious compartment, and p is the AR. Except for AR and transmissibility rate, parameters can be set as fixed for the dominant virus variant. Due to changing testing and tracing strategies, people's compliance to report their risk contacts, etc., AR $p = p(t)$ is time-varying and specific both in space and time since each country has its own testing strategy and report. Specific ZSEIAR model presented in the next subsection used to model the spread of COVID-19 in the Czech Republic is an aggregate mechanistic model with a very low number of compartments, but still sufficient to accurately describe the dynamics of the epidemic in all compartments.

Comparison of SEIAR and SEIR models

The standard SEIR model is included in SEIAR model type for $p = 1$. The non-epidemic equilibrium of the system (1) becomes unstable when the basic reproduction number

$$R_0 := \frac{\beta p}{\mu_1} + \frac{\beta(1-p)}{\mu_2} \tag{2}$$

exceeds one. You can see that it can be interpreted as a weighted average of reproduction numbers of both the infectious compartments I and A. This is in accordance with standard R_0 definition in case of SEIR model (1) for $p = 1$. This claim can be proved using The Jacobian linearization matrix of the system (1)

$$J = \begin{pmatrix} -\beta(I+A) & 0 & -\beta S & -\beta S & 0 \\ \beta(I+A) & -\gamma & \beta S & \beta S & 0 \\ 0 & \gamma p & -\mu_1 & 0 & 0 \\ 0 & \gamma(1-p) & 0 & -\mu_2 & 0 \\ 0 & 0 & \mu_1 & \mu_2 & 0 \end{pmatrix}$$

evaluated in the non-epidemic equilibrium $(1, 0, 0, 0, 0)$ as

$$J(1,0,0,0,0) = \begin{pmatrix} 0 & 0 & -\beta & -\beta & 0 \\ 0 & -\gamma & \beta & \beta & 0 \\ 0 & \gamma p & -\mu_1 & 0 & 0 \\ 0 & \gamma(1-p) & 0 & -\mu_2 & 0 \\ 0 & 0 & \mu_1 & \mu_2 & 0 \end{pmatrix}. \tag{3}$$

The non-epidemic equilibrium loses stability if an eigenvalue of a submatrix

$$A = \begin{pmatrix} -\gamma & \beta & \beta \\ \gamma p & -\mu_1 & 0 \\ \gamma(1-p) & 0 & -\mu_2 \end{pmatrix}$$

crosses imaginary axes and becomes positive in its real part. The characteristic polynomial of the matrix A is

$$\begin{aligned} p(\lambda) = \quad & \lambda^3 + (\gamma + \mu_1 + \mu_2)\,\lambda^2 + (-\beta\,\gamma + \gamma\,\mu_1 + \gamma\,\mu_2 + \mu_1\mu_2)\,\lambda + \beta\,\gamma\,p\mu_1 - \\ & -\beta\,\gamma\,p\mu_2 - \beta\,\gamma\,\mu_1 + \gamma\,\mu_1\mu_2 \end{aligned}$$

and Routh-Hurwitz criterion

$$\begin{aligned} \gamma + \mu_1 + \mu_2 \quad &> \quad 0, && (4) \\ -\det A = \beta\,\gamma\,p\mu_1 - \beta\,\gamma\,p\mu_2 - \beta\,\gamma\,\mu_1 + \gamma\,\mu_1\mu_2 \quad &> \quad 0, && (5) \\ (\gamma + \mu_1 + \mu_2)\,(-\beta\,\gamma + \gamma\,\mu_1 + \gamma\,\mu_2 + \mu_1\mu_2) - & && \\ -\,(\beta\,\gamma\,p\mu_1 - \beta\,\gamma\,p\mu_2 - \beta\,\gamma\,\mu_1 + \gamma\,\mu_1\mu_2) \quad &> \quad 0, && (6) \end{aligned}$$

implies negative real parts of all the eigenvalues of the matrix A. The condition (4) is always satisfied. Violation of condition (5) implies that at least one eigenvalue has a positive real part. Condition (5) can be equivalently rewritten as $R_0 < 1$, and if $R_0 > 1$, then epidemic outbreaks occur. Violation of condition (6) gives rise to an unstable focus (i.e., two complex eigenvalues crossing the imaginary axis).

Important assumptions of mixing and low level of reinfections in SEIR-type models

There are few often neglected but essential assumptions that should be satisfied for epidemic modeling, scenario development and model calibration. In addition to the good and possibly time-varying estimation of the model parameters, it is first of all the basic assumption of random homogeneous mixing - i.e. the existence of persistent or frequent risk contacts. However, such an assumption is not satisfied in an outbreak of a new respiratory disease or

in the case of various NPIs such as lockdowns or school closures. Since many infectious people are asymptomatic or presymptomatic, a proportion of the infected population is also not detected, so we do not know how much of the population may encounter infectious people. However, it is the size of the population that may have frequent risk contacts with the infected (referred to as clusters) that scales the size of compartments in SEIR-type models. There is a possibility to use agent-based modeling and simulate epidemic dynamics on a contact network, using social contact matrices, but these models are limited by, e.g., the need to create a multi-layered contact structure for a realistic model. Even then, agent-based models are capable of simulating a town of 56,000 [6] with almost 3 million contacts, but not an entire country. In the case of the COVID-19 spread model in the Czech Republic, we therefore decided to use a compartmental continuous-time model ZSEIAR where this contact-risk population is not fixed.

Another important assumption for using the SEIR-type model is the low number of reinfections. Reinfections were negligible even in time of delta variant of SARS-CoV-2. From the beginning of the year 2022 when the omicron variant started to spread, the SEIARS model had to be used with an additional estimated parameter that describes the flow from the compartment R (removed) back to S (susceptibles).

Important assumptions on ascertainment rate computation validity

There are important assumptions for using the method of AR computation described in the main paper. Primarily, the aggregate number of hospitalizations within a 7-day or 14-day moving interval must be sufficiently large to ensure an adequate sample size in each interval. Given that the AR is calculated as a ratio, it is imperative that the denominator is neither zero nor a diminutive figure, as this leads to an inflated variance and consequently, a biased result. In instances where these conditions are unmet, one must opt for either an extended temporal interval or an enlarged spatial region.

Provided there is an adequate count of hospital admissions, the proposed methodology can be applied at a regional level[1]. Figure 1 illustrates the period from April 4, 2020 to July 26, 2020, during the outbreak in the Moravian-Silesian region of the Czech Republic. It becomes apparent that the regional computation holds validity only during the outbreak surge in July, and it naturally yields a higher estimate of the AR than the estimate calculated using data from the entirety of the Czech Republic. During this time frame, the accuracy of the estimate is inherently improved. Conversely, a dearth of hospitalizations during the month of May amplifies the variance.

Figure 1 shows that there was indeed an increase in AR during the period of area testing in this region and thus it explains a disproportion between new cases and hospitalizations even better than in case of usage the full dataset.

[1] Due to the inflated variance the AR may exceed 1, in such case we set the AR equal to 1.

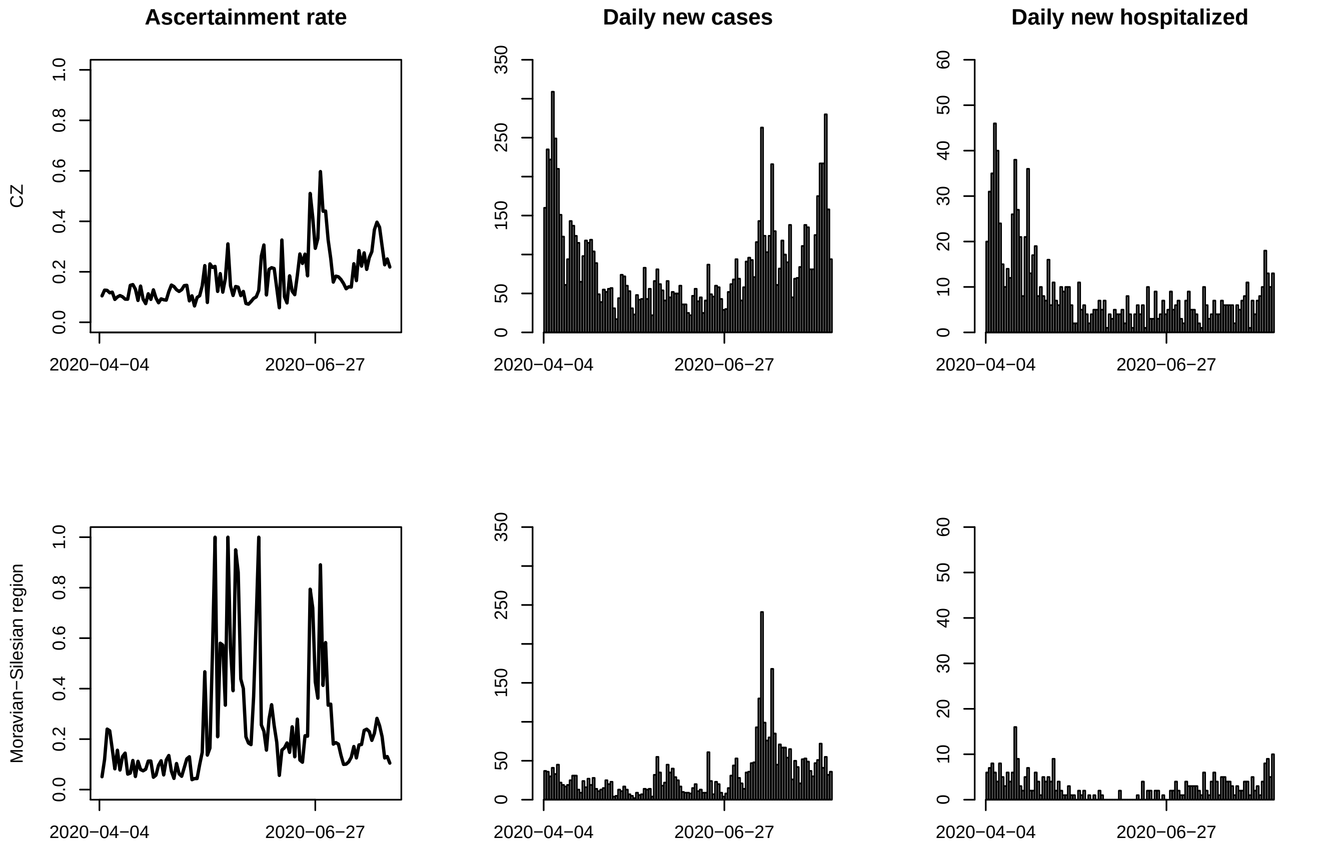

Fig 1. Comparison of the AR of the whole Czech Republic and of the Moravian-Silesian region. The top row shows figures related the whole country, the bottom row shows figures for the Moravian-Silesian region. In the first column you can see the AR computed in both cases from 7-days moving averages, in the second column you can see number of daily new cases, and in the third column number of daily new hospitalized.

Specific model ZSEIAR

We present our compartmental model ZSEIAR in the form

$$\begin{aligned}
\dot{Z} &= -\varepsilon Z/N, \\
\dot{S} &= -\tfrac{\beta}{N-Z} S(I+A) + \varepsilon Z/N, \\
\dot{E} &= \tfrac{\beta}{N-Z} S(I+A) - \gamma E, \\
\dot{I} &= \gamma p E - \mu_1 I, \\
\dot{A} &= \gamma(1-p)E - \mu_2 A, \\
\dot{Q} &= \mu_1 I - \nu Q, \\
\dot{R} &= \nu Q,
\end{aligned} \tag{7}$$

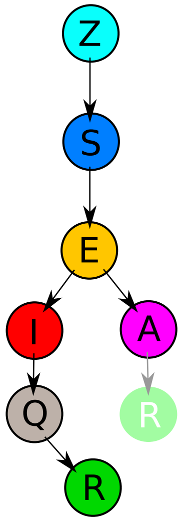

Z not affected population size
S susceptibles
E exposed
I detected infectious
A undetected infectious
Q isolated infectious
R removed detected
N population size
p ascertainment rate
$\varepsilon, \beta, \gamma, \mu_1, \mu_2, \nu$ parameters

The model (7) has an additional compartment Z of people with a negligible risk of contact with infectious and compartment Q of isolated reported positives. The compartment R of removed is also set as the detected removed for real data comparison purposes. Most of the parameters are time-dependent. All variables and parameters are described in Table 1 and more detailed information about parameter calibration, estimation, optimization, and dataset relations is described below. We enclose the R-code [7, 8]. The compartments in the model, including the AR $p(t)$ estimate, should be related to the date of the positivity report of the person to explain the visible part of the epidemic and the induced variables.

There are two specificities of the model (7) that are important and novel: (i) the model estimates the AR independently as a 7-days (possibly 14-days) moving average, so it is not calibrated or fixed and the method is fully described in the main article, (ii) the model estimates the affected cluster size S of susceptible people by optimization to the complement compartment Z and so an aggregate approach of homogeneous mixing can be used within the susceptible population S. Compartment S of affected clusters for which the assumption of random homogenous mixing is fulfilled has unknown size. We can estimate this unknown size of affected clusters by optimizing them to compartmental time series, whereas the other parameters have to be estimated differently (from data, literature, information from outbreaks in other countries, etc.). The complement of S in the entire monitored population is compartment Z, so it can be assumed that Z is the compartment of people with negligible risk of contact with infectious. Due to this method of defining Z there is no need to change transmissibility rate parameter β according to NPIs, season, etc. and it can be set either to a constant or solely dependent on mobility as we did. This allows for a plausible usage of the model also in the case of COVID-19 epidemic outbreak that came into the naive population. Throughout the whole time, we may assume that there is a relatively high risk of being infected through the contact social network within the affected clusters and, on the contrary, the compartment Z is far enough away from the social network to avoid contact with the infected within the affected clusters. From this point of view, the compartment Z of model (7) substitutes the network structure in the agent-based modeling approach in the most simple way. If the disease is already widespread in the population, we can set $Z = 0$, which leads to a standard SEIR model.

Label	Description	Source	Computation
N	population	parameter	fixed, $N = 10^7$
$Z = Z(t)$	unaffected population at time t, clusters out of the risk to meet infectious	state variable	Runge-Kutta method, initial condition $Z(0) = N - S(0)$
$S = S(t)$	susceptible population at time t, clusters at the high risk to meet infectious	state variable	Runge-Kutta method, initial condition $S(0) = 10^4$
$E = E(t)$	exposed population at time t, infected in incubation period, not infectious; latency period	state variable	Runge-Kutta method, initial condition $E(0) = 0$
$I = I(t)$	infectious reported population at time t	state variable	Runge-Kutta method, initial condition $I(0) = 0$
$A = A(t)$	infectious unreported population at time t	state variable	Runge-Kutta method, initial condition $A(0) = 10^{-3}$
$Q = Q(t)$	non-infectious or isolated infectious reported population at time t	state variable	Runge-Kutta method, initial condition $Q(0) = 0$
$R = R(t)$	reported recovered or deceased at time t	state variable	Runge-Kutta method, initial condition $R(0) = 0$
$\varepsilon = \varepsilon(t)$	rate of cluster growth at time t	parameter	optimized to the hospitalized (LSM) or exposed, respectively, using the age-dependent estimation of $P(H)$ and the AR estimate
$p = p(t)$	AR at time t	parameter	estimated, see this paper
$\beta = \beta(t)$	transmissibility rate at time t	parameter	calibrated, strictly dependent on the average number of contacts or mobility, respectively
$\gamma = 1/4$	reciprocal of the mean non-infectious incubation period	parameter	fixed, incubation period is 5-6 days, contagiousness onset is 1-2 days before symptoms onset [9, 10]
$\mu_1 = \mu_1(t)$	reciprocal of the mean infectious period of the reported	parameter	computed from reported data, for more details see the next section
$\mu_2 = 1/3$	reciprocal of the mean infectious period of the unreported	parameter	calibrated and fixed
$\nu = \nu(t)$	reciprocal of the mean quarantine duration	parameter	computed from reported data, for more details see the next section

Table 1. Model (7) description and the calibration of parameters.

Parameters of the model (7) are calibrated or estimated and consequently optimized to fit all compartments by optimizing the compartment Z. Varying parameter ε in time determines Z together with all other compartments to get the mean single model fit and the mean forecast scenario keeps the last value of ε. We observed that during epidemic outbreaks, the optimally calibrated ε was increasing, which is consistent with the idea of cluster growth. Similarly, a significant decrease in the growth of the affected cluster was timely, consistent with NPIs such as mandatory use of FFP2 respirators, school closures, etc. (see Subsection Ascertainment rate estimate usage -– effectiveness of NPIs)

The primary goal of the specific ZSEIAR model was to provide possible scenarios for the occupancy of hospital beds during the COVID-19 epidemic in the Czech Republic (software tool Monitoring, analysis, and management of epidemic situations, [11]). We combine the possible scenarios with data from the Czech National Control Centre for Intensive Care to provide supporting material for the government decision-making process. The model played a crucial role in the decision-making process, especially around the peak of the epidemic, when the pressure on hospitals was the largest. Nevertheless, the model (7) as it is cluster-optimized, is specific for the Czech Republic. For other countries/communities it has to be re-optimized by finding a specific time series ε.

Data sources and parameter estimates

Data on reported SARS-CoV-2 positive individuals and patients with COVID-19 and their hospital stays are collected and processed by the Information System of Infectious Diseases (ISID) almost in real-time [12]. From the data set [13] provided by The Institute of Health Information and Statistics of the Czech Republic (IHIS), we continuously estimated the mean times in individual compartments and the AR estimate. ISID includes a complete record of health care information about a person. We used a dataset that includes variables describing the infection case: district and regional number, sex, age group (0-19, 20-64, 65-), date of the first symptom, date of sampling collection, date of positive result, date of report, date of isolation, date of admission to a hospital, end of hospitalization, date of recovery, date of death. Additional useful information can be mined from this selected dataset, as mean duration from symptoms to report, from report to hospital admission or to death, etc.

Parameter γ estimate

Parameter γ is the reciprocal value of the mean incubation time, which represents the time interval between the time of infection and the time when the organism becomes infectious to its surroundings. According to standardly used sources such as [9, 14–17] virus transmissibility is a common assumption even in the pre-symptomatic period. In the model, we use a mean incubation latency period of 4 days. The length of the incubation period does not have a significant effect on the modeled and predicted quantities, because the model does not scale in terms of the height of the epidemic peak.

Estimates of parameters μ_1 and μ_2

Parameters μ_1 and μ_2 are the reciprocal values of the mean duration of the infectious period of subjects positively diagnosed with the presence of SARS-CoV-2 $1/\mu_1$), $1/\mu_2$ is the mean infectious period for infected unreported subjects. The duration of infectiousness is very variable and probably depends on many circumstances (initial dose of the virus, age, immune response of the organism, prevalence in locality, etc.). Interesting is that the mean viral shedding period of SARS-CoV-2 is even 4.76 days according to ECDC [10], but the common estimate of the infectious period in the modeling community is 3 or 4 days [18, 19], the short-term IHIS model for the Czech Republic [12, 20] uses an average length of infectiousness of an individual of 4 days, a long-term model of a scientific team around Richard Neher (Biozentrum, University of Basel) uses the default value of 3 days [18], some case studies use even less than 2 [21]. During model calibration, we tried $1/\mu_2$ from 3 to 6.3 days according to the literature, for example [9, 14–17] (with complementary recalibrated β), and the best performance appears to be for $1/\mu_2 = 3$. The explanation may be that PCR positivity is not a necessary condition for an individual's infectivity, asymptomatic undetected subjects can have lower viral shedding, the symptomatic subjects self-isolate after the symptoms onset more often, or the amount of respiratory virus shed decreases over time. Another explanation is that our model

assumes a higher probability of encountering an infectious person because it works within affected clusters and these two parameters are complementary in the definition and principle of R_0.

To estimate parameter μ_1 from the data, we used the mean period until the isolation of the individual. The value $1/\mu_1$ is equal to the minimum of the mean period until isolation and the mean length of infectiousness. If tracing and testing were greatly accelerated (e.g., through community antigen testing in workplaces and schools), the mean period of isolation time could fall below this mean time, and this could make a significant contribution to reducing the stress on hospitals. The mean period until isolation was calculated as 2 plus the 7-day moving average of the difference between the time of isolation and the first symptoms and is related to the day of the report [2]. This value can also be used, among other things, for an early warning system (if it starts to grow, it means that the tracing system is overfull) or for comparing the situation in specific regions. It was used during the second half of 2020 for regional comparisons, and the resulting parameter was published on the website [20].

Parameter ν estimate

The mean time to be removed from active cases is the reciprocal value of the ν parameter, and we do not distinguish between recovered and dead. Because the compartment R serves only as a supporting variable, this parameter is not very significant.

The value $1/\nu$ is estimated from the IHIS data set, which contains anonymized data on individual positively diagnosed subjects, as the median number of days from the date of the report to recover or death. The median is calculated as a 7-day moving median after the reporting date. If less than half of the people are removed in a given period, the value is set to 10 days (the typical quarantine duration in the monitored period).

Parameter β estimate

We estimated the parameter β during the first outbreak. After the closure of schools 2020/03/11 and the announcement of the state of emergency on 2020/03/16, there was a consequent reduction in the number of contacts in the population. The fit of the parameter β during the period of the first outbreak corresponded to a decrease in contacts by a third and a slow increase, which is correlated with the results of the ongoing study by Daniel Prokop published on [22], so we started to use data from this sociological study as an estimate of the number of contacts on which β depends. Because the number of social contacts reported in the study is highly correlated with data on population mobility [23], we are using mobility data to estimate β now (see Figure 2).

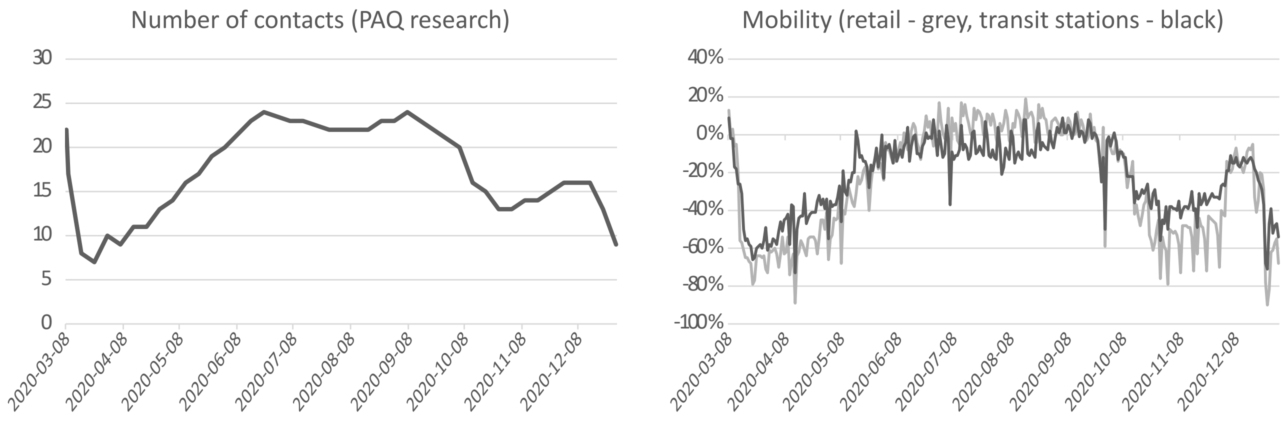

Fig 2. Number of contacts and mobility. The usual number of visits and length of stay (left, [22]) compared to mobility percentage increase or decrease (right, [23], [24]) during the COVID-19 epidemic in the Czech Republic, 2020.

[2] Of course, after omitting meaningless data, we do not assume an infectious period is longer than 14 days.

In our model, the β parameter is only affected by the number of contacts, $\beta = 19/220\times$ number of contacts per week[3] reported in [22] or an LSM estimate (for 2020 dataset) of the same that is 1.68 increased by 0.0197 times the average mobility from the weekly reported mobility in the Czech Republic according to the mobility report [23], other dependencies we have moved to the optimized parameter ε (e.g. difference between winter and summer, possibility of infection across borders), which affects the size of the susceptible compartment S.

Parameter p estimate

Moving AR estimate p calculation is described in detail in the main part of the paper. We use the anonymized data set, available at [13], provided by IHIS for predictive models.

Optimization

For the correct calculation of parameters from the data set provided by IHIS for predictive models, we exclude from the calculations of the parameters p, μ_1 and ν some incomplete data (lines) due to the fact that they are in some way incorrect. The period from first symptoms to isolation must be between -2 and 14, the duration of the recovery period must be positive or not specified, and the duration of the period to death must be positive or not specified.

Our model does not assume that the virus weakened in the summer (virulence has not decreased), but the viral load in the population has decreased, probably due to the weather and NPIs in Europe.

After all the previously described estimations and calibration, the model can be optimized continuously during time to hospitalized or exposed, respectively, by calibrating affected susceptible clusters S using $\varepsilon(t)$. We assume that the number of people admitted to hospital is given by a the probability of hospitalization $P(H)$ from the compartment E of exposed individuals. In the main paper we derived our estimate

$$P(H) = \left(1 + 11p^{+}_{65+}\right)P(H_{65-}),$$

where p^{+}_{65+} is a 7-day moving average of the senior population ratio in the reported cases and probability of hospitalization of infected under 65 aged subject is set to $P(H_{65-}) = 1/160$. This assumption is confirmed by the fit to the real hospitalization incidence during the second outbreak in autumn 2020 (using LSM) $H(t) \sim aE(t)$, where $E(t)$ is the estimate of the number of persons exposed in the compartment (people during the incubation period) on the day t and $H(t)$ is the number of daily new hospitalized people with the corresponding delay (both the hospitalized and the estimate of the number of exposed are monitored according to the same date of report).

We optimized ε in the first epidemic period before the second wave (till autumn 2020) in optimally selected values (beginning and end of the emergency state, area testing in OKD, etc.), and at the onset of the second wave the optimization of ε revealed an exponential growth in increasing cluster size (doubling time about 10 days). After introducing NPIs on 22.10., there is an evident decrease in ε and consequently, that implied deceleration of the size of the affected clusters increase (resulting from the performed data optimizations). We optimized the Christmas 2020 period retrospectively, with a large variance in the measured data due to free antigen testing and public holidays, which we smoothed out with a 21-day kernel estimate.

Due to the data revisions (lately reported hospitalizations and deaths, for example), the optimization is computed for the last month to reload the revised and corrected data, while a month or older data are already considered as given and the optimization is taken as it is (computed in advance) to make the calculation less time consuming.

The previously optimized values of ε of the last month are re-calibrated in the 5-day interval by selecting the smallest deviation of the time series $E(t)$, which is calculated by the model with the estimated AR. Thus, we compare the compartment of exposed individuals in the model for various parameters ε close to the previous one (80 - 120 %) with the estimated real compartment of exposed persons with knowledge of the AR. The real exposed

[3] The highest average number of risk contacts per week were 22 people, so the basic reproduction number for the wild variant in 2020 corresponding to our estimate is $\beta = 19/220 \times 22$ mean infectious time $= 5.7$ that is possible according to meta-analysis [25]. We have to point out that the basic reproduction number depends obviously on factors such as the viral load in the community or the type of the community, etc.; for example, the highest reproduction number was estimated for Diamond Princess Cruise Ship in Japan as 14.8. Even our fairly high estimate for the wild SARS-CoV-2 variant is due to the fact that we are working with the compartment S of affected clusters. If we performed the calculation on the whole population, R_0 would probably be lower.

compartment consists of reported newly infected divided by p and multiplied by 4 days, the mean duration in the compartment. Thus, we obtain an estimate of all newly infected from the observation layer. Due to weekly fluctuations in reporting, we use a seven-day moving average. All calculations are related to the date of reporting, so they are consistent over time. However, the optimization can also be performed analogously on data of hospitalized subjects or data of new cases, since we assume $H(t)$ is proportional to $E(t)$ and the AR gives information about observed new cases.

Due to its principles, the model has a limited long-term prediction ability, but a very good ability to monitor the epidemiological situation, as well as the possibility to partially monitor, for example, differences in individual regions using p. By optimizing virus-exposed individuals, the short-term prediction of the number of hospitalized and deaths (10-14 days, deaths up to 3 weeks) is very good, assuming stable virulence. Therefore, the model was used during 2020 and 2021 as a supporting model for monitoring and estimating the capacity of the health care system (especially ICUs capacities) through Czech application [11] for Monitoring, Analysis and Management of Epidemic Situations that was developed together with IHIS and two departments of Masaryk University (Intitute of Biostatistics and Analysis and Department of Mathematics and Statistics), Brno.

Long-term prediction is in principle impossible for an epidemic controlled by the social behavior of people, NPIs, efficiency of tracing, etc. However, a retrospective analysis of the optimalization results with respect to NPIs can show how successful the NPIs were and what effect they have had. Basic results can be seen in a glance as you can see in the main paper (Figure 7).

References

1. Walsh KA, Jordan K, Clyne B, Rohde D, Drummond L, Byrne P, et al. SARS-CoV-2 detection, viral load and infectivity over the course of an infection. Journal of Infection. 2020;81(3):357–371.
2. Gao M, Yang L, Chen X, Deng Y, Yang S, Xu H, et al. A study on infectivity of asymptomatic SARS-CoV-2 carriers. Respiratory medicine, 169, 106026.
3. Hu Z, Song C, Xu C, Jin G, Chen Y, Xu X, et al. Clinical characteristics of 24 asymptomatic infections with COVID-19 screened among close contacts in Nanjing, China Science China Life Sciences. 2020;1—6.
4. Zou L, Ruan F, Huang M, Liang L, Huang H, Hong Z, et al. SARS-CoV-2 viral load in upper respiratory specimens of infected patients. New England Journal of Medicine. 2020;382(12):1177—1179.
5. Pullano G, Di Domenico L, Sabbatini CE, Valdano E, Turbelin C, Debin M, et al. Underdetection of cases of COVID-19 in France threatens epidemic control. Nature. 2021;590(7844):134—139.
6. Berec L, Diviák T, Kuběna A, Levínskỳ R, Neruda R, Suchopárová G et al. On the contact tracing for COVID-19: A simulation study Epidemics. 2023;43:100677.
7. R Core Team. R: A Language and Environment for Statistical Computing. `https://www.R-project.org/`. 2021.
8. RStudio Team. RStudio: Integrated Development Environment for R. `http://www.rstudio.com/`. 2020.
9. Lauer SA, Grantz KH, Bi Q, Jones FK, Zheng Q, Meredith HR, et al. The incubation period of coronavirus disease 2019 (COVID-19) from publicly reported confirmed cases: estimation and application. Annals of internal medicine. 2020;172(9),577–582.
10. ECDC. COVID-19 Infection. `https://www.ecdc.europa.eu/en/covid-19/latest-evidence/infection`. 2021.
11. Pavlík T, Komenda M, Přibylová L, Uher M, Májek O, Kraus A, et al. MAMES – Monitoring, analysis, and management of epidemic situations; `https://webstudio.shinyapps.io/MAMES/`. 2020.
12. Komenda M, Bulhart V, Karolyi M, Jarkovský J, Mužík J, Májek O, et al. Complex reporting of the COVID-19 epidemic in the Czech Republic: Use of an interactive web-based app in practice. Journal of medical Internet research. 2020;22(5):e19367.

13. Institute of Health Information and Statistics of the Czech Republic, Ministry of Health of the Czech Republic. Open Data Sets CZ COVID-19. https://onemocneni-aktualne.mzcr.cz/api/v2/covid-19. 2020.

14. Guan Wj, Ni Zy, Hu Y, Liang Wh, Ou Cq, He Jx, et al. Clinical characteristics of coronavirus disease 2019 in China. New England Journal of Medicine. 2020;382(18),1708–1720.

15. Li Q, Guan X, Wu P, Wang X, Zhou L, Tong Y, et al. Early transmission dynamics in Wuhan, China, of novel coronavirus–infected pneumonia. New England Journal of Medicine. 2020;382:1199–1207

16. Kucharski A, Russell T, Diamond C, Liu Y. Analysis and projections of transmission dynamics of nCoV in Wuhan. CMMID repository. 2020;2.

17. WHO, Joint Mission. Report of the WHO-China Joint Mission on Coronavirus Disease 2019 (COVID-19). https://www.who.int/docs/default-source/coronaviruse/who-china-joint-mission-on-covid-19-final-report.pdf. 2020.

18. Neher R, Aksamentov I, Nol N, Albert J, Dyrdak R. COVID-19 Scenarios. urlhttps://covid19-scenarios.org/. 2020.

19. Ibrahim MA, Al-Najafi A. Modeling, control, and prediction of the spread of COVID-19 using compartmental, logistic, and gauss models: a case study in Iraq and Egypt. Processes. 2020;8(11):1400.

20. Institute of Health Information and Statistics of the Czech Republic, Ministry of Health of the Czech Republic. Reports and methodics. https://onemocneni-aktualne.mzcr.cz/covid-19/zpravy-a-metodiky. 2020.

21. Kumari P, Singh HP, Singh S. SEIAQRDT model for the spread of novel coronavirus (COVID-19): A case study in India. Applied Intelligence. 2021;51(5):2818—2837.

22. PAQ research, IDEA AntiCovid. Life in pandemic: How many people were the respondents in closer contact with? https://zivotbehempandemie.cz/kontakty. 2020.

23. Google. Google Mobility reports. https://github.com/ActiveConclusion/COVID19_mobility/blob/master/google_reports/mobility_report_europe.xlsx. 2020.

24. Google. Mobility Report CSV Documentation. https://www.google.com/covid19/mobility/data_documentation.html?hl=en. 2020.

25. Billah MA, Miah MM, Khan MN. Reproductive number of coronavirus: A systematic review and meta-analysis based on global level evidence. PloS one. 2020;15(11):e0242128.
